# One for All—A Highly Efficient and Versatile Method for Fluorescent Immunostaining in Fish Embryos

**DOI:** 10.1371/journal.pone.0019713

**Published:** 2011-05-13

**Authors:** Daigo Inoue, Joachim Wittbrodt

**Affiliations:** 1 Centre for Organismal Studies, University of Heidelberg, Heidelberg, Baden-Württemberg, Germany; 2 Institute of Toxicology and Genetics, Karlsruhe Institute of Technology, Eggenstein-Leopoldshafen, Baden-Württemberg, Germany; VIB & Katholieke Universiteit Leuven, Belgium

## Abstract

**Background:**

For the detection and sub-cellular (co)-localization of proteins in the context of the tissue or organism immunostaining in whole mount preparations or on sections is still the best approach. So far, each antibody required its own fixation and antigen retrieval protocol so that optimizing immunostaining turned out to be tedious and time consuming.

**Methodology/Principal Finding:**

Here we present a novel method to efficiently retrieve the antigen in a widely applicable standard protocol, facilitating fluorescent immunostaining of both cryosections and whole mount preparations in zebrafish (*Danio rerio*) and medaka (*Oryzias latipes*).

**Conclusions/Significance:**

Our method overcomes the loss of sections and damage of tissue and cell morphology, and allows parallel immunostaining in multiple colors, co-immunostaining with fluorescent proteins in transgenic fish lines and in combination with whole mount *in situ* hybridization.

## Introduction

Analyses of expression and localization of proteins are crucial to determine the function of genes at tissue and organ levels mainly by immunohistochemical and live-imaging analyses. While *in vivo* imaging of exogenous fusion proteins allows to grasp their spatio-temporal expression and localization, it is important to validate the expression and sub-cellular localization of endogenous proteins. This is best analyzed by immuno-histochemistry in sections or whole mount preparations. In principle, immunostaining is easy to perform, however, achieving the optimal immunostaining for each antibody demands various types of modifications of the protocol (e. g. choice of fixatives and retrieving antigens with appropriate buffers [1], [2]). Those modifications for the step-wise improvement of immunostaining are very time and reagent consuming. Key step in the improvement of immunostaining is the efficient retrieval of the antigen, by which the antibody can access its corresponding epitope blocked mainly by artificial protein cross-linking during fixation [1], [2]. To retrieve the antigen after fixation, samples are either treated with enzymes or heated in appropriate buffers [1], [2]. Even though these approaches enhance the signal of stainings, they often cause damage and detachment of the samples. It has been a long-standing aim in life sciences to establish a universal method for immunostaining with efficient and reproducible antigen retrieval [3], [4] as we present here.

## Results and Discussion

### Efficient improvement of fluorescent immunostaining for cryosections by a novel heating method

**Figure 1:**
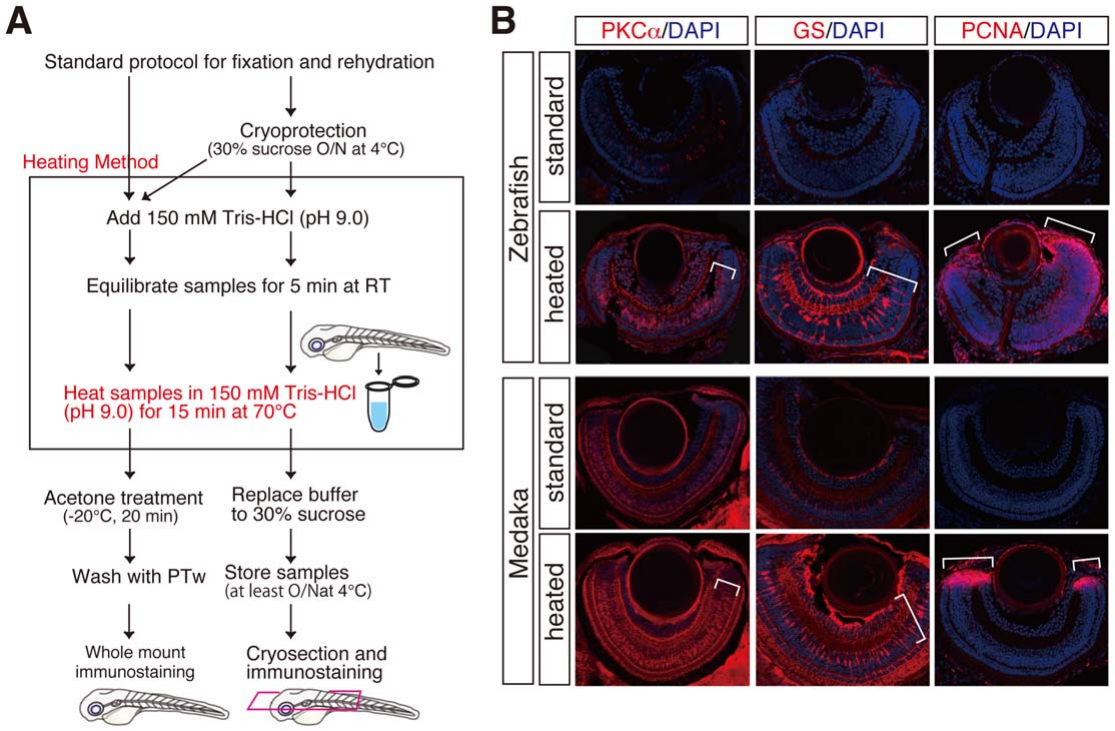
Improvement of fluorescent immunostainings by a novel heating method. (**A**) Schematic flowchart including a novel heating step in the standard immunostaining for cryosections and whole mount preparations. Whole mount and cryoprotected fish embryos were heated at 70°C for 15 min in 150 mM Tris-HCl at pH 9.0 (framed), subjected to the respective standard protocol for immunostaining. Cryoprotected preparations also can be used for whole mount immunostaining (bifurcated arrows). (**B**) Fluorescent immunostainings with cryosections of zebrafish and medaka retinae using anti-PKCα, anti-GS and, anti-PCNA antibodies. PKCα, GS, and PCNA immunostainings mark bipolar, Mueller glia, and retinal progenitor cells, respectively. Note that the heating method (heated) but not standard method (standard) efficiently improved all the immunostainings (lower panels, see brackets). Nuclei were counterstained with DAPI (blue).

To carefully assess the improvement of immunostaining and maintenance of tissue integrity, we chose the highly ordered neural retina of zebrafish and medaka which allowed the use of specific antibodies for neuronal and stem cells to analyze proliferation and differentiation of neural progenitor cells [3], [5]. In a first step, we approached immunohistochemistry on cryosections that are not only expeditious but also preserve the physiological epitope better than plastic sections [3], [6]. We aimed at retrieving the antigens prior to sectioning not to additionally damage the fragile section with the antigen retrieval procedure. We efficiently retrieved the antigen by complementing the standard immunostaining protocol for fish by a novel heating-step (Figure 1A and Materials and Methods). In this step, we used Tris-HCl at pH 9.0 as an antigen retrieval buffer for efficient pH-dependent antigen retrieval [7], [8]. After determining the buffer concentration (see details in Materials and Methods) that efficiently preserves the morphology, we heated entire embryos at 70°C for 15 min, and then re-cryoprotected them in 30% sucrose at 4°C overnight (Figure 1A). In both, zebrafish and medaka, the heating method did not affect the morphology of the embryo in general and the highly ordered retina in particular more than untreated controls. Since the heating step is applied to whole mount preparations prior to sectioning, the loss of sections during antigen retrieval is not an issue. In all immunostainings under the universal conditions established here, we used a fixed dilution rate of 1∶500 for all the antibodies applied.

**Figure 2:**
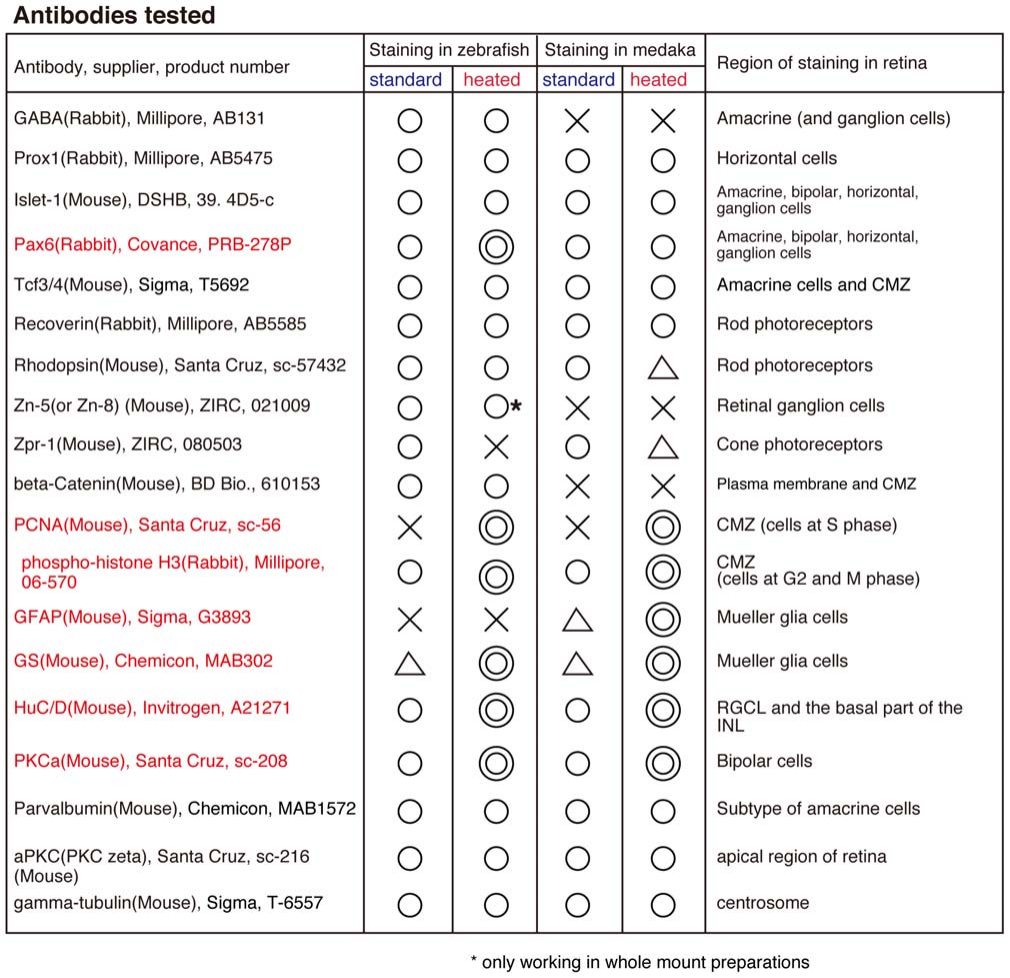
Antibody list tested in zebrafish and medaka with or without the heating method. The efficacy of immunostaining of cryosections based on the fluorescent intensity is indicated by double circled, circled, triangle, crossed. Double circled; working well. Circled; working. Triangle; weak. Crossed; not working. Most immunostainings with the heating method were comparable to those with the standard protocol except for the immunostaining of Zpr1, maybe due to the masking of the antigens by heating. Importantly, the heating method appreciably improved some immunostainings, which are important markers of neuronal and progenitor/stem cells. CMZ; ciliary marginal zone, RGCL; retinal ganglion cell layer, INL; inner nuclear layer.

To test the efficacy of our new method, we first focused on antibodies that at the given dilution in the standard protocol only gave a weak or no signal (Glutamine synthetase (GS), PKCα, and PCNA) in both zebrafish and medaka (Figure 1B, upper panels). Using the heating method remarkably improved GS, PKCα, and PCNA immunostainings that resulted in robust fluorescent signals and clearly marked Mueller glia, bipolar and proliferating progenitor cells respectively (Figure 1B, see the brackets at the lower panels). In addition, the heating method often even improved immunostainings for antibodies, which worked in the standard protocol in either zebrafish or medaka (Figure S1 and Figure 2). These results clearly demonstrate that the heating step we introduced efficiently retrieves the antigens for immunostaining on cryosections without losing or damaging samples.

### Application of the heating method to multiple fluorescent immunostaining of cryosections and whole mount embryos

**Figure 3:**
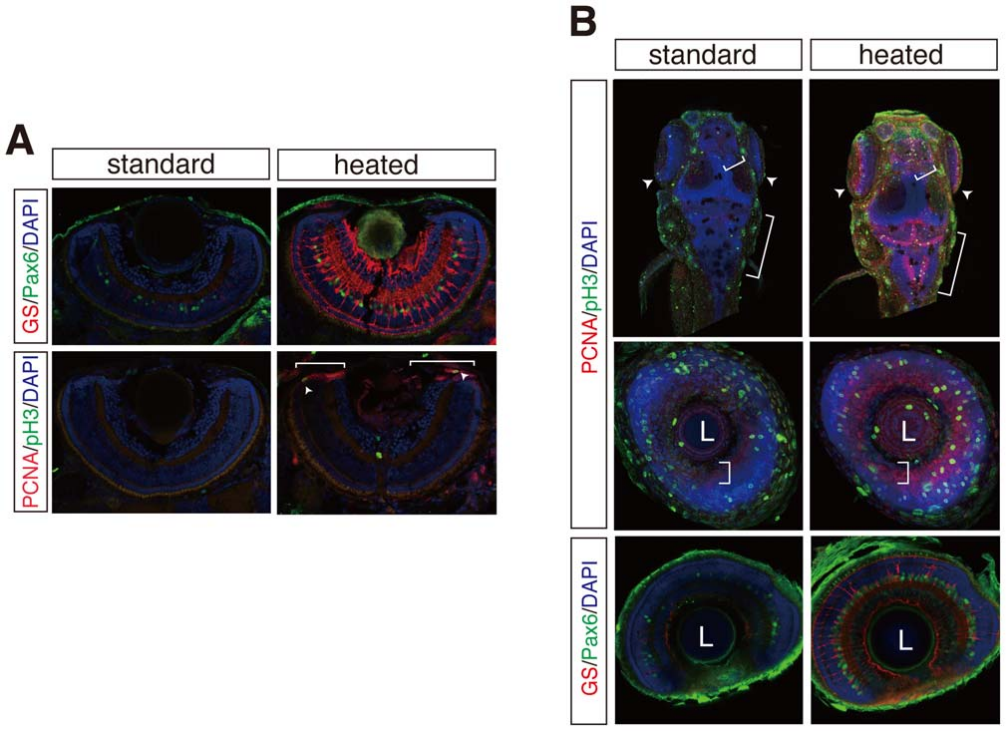
Multiple fluorescent immunostaining of cryosections and whole mount embryos by the heating method. (**A**) GS/Pax6 (top panels) and PCNA/phospho-histone H3 (pH 3) (bottom panels) immunostainings on cryosections of zebrafish retinae. GS and Pax6 immunostainings detect amacrine and Mueller glia cells. pH 3-positive mitotic cells (arrowheads) co-localize to PCNA-positive retinal progenitor cell region (brackets) in the ciliary marginal zone (CMZ). (**B**) Whole mount fluorescent immunostainings as in **A** with or without the heating method. Maximum projection of dorsal view of whole mount zebrafish (top panels). Coronal optical sections of zebrafish retina (middle and bottom panels). Brackets and arrowheads indicate proliferative zones of hindbrain, optic tectum, telencephalon, and CMZ. L; lens. Nuclei were counterstained with DAPI (blue).

Given the remarkable improvement of GS and PCNA immunostainings (Figure 1), we used these antibodies to assess the efficacy of the heating method in other applications such as multicolor immunostainings. Since the optimization of the multi-color immunostaining protocol is further limited by the use of different antibodies [9], we first tested whether our procedure can be applied using the universal conditions as in Figure 1 for multiple antibodies. Immunostainings with the heating method resulted in clear and strong staining of both, Pax6-positive amacrine cells and GS-positive Mueller glia cells, while the standard protocol under the same conditions resulted only in a weak staining of few Pax6-positive amacrine and GS-positive Mueller glia cells (Figure 3A, top panels). Similarly, double immunostainings with the heating method, but not with the standard protocol, using anti-phospho-histone H3 (pH 3) antibody and anti-PCNA antibody clearly delineated the progenitor-cell region at the ciliary marginal zone (CMZ) by staining proliferating (PCNA) and dividing (pH 3) retinal progenitor cells (Figure 3A, bottom panels). This approach was also successful in medaka with the same antibodies (and concentration) in both GS/Pax6 and PCNA/pH 3 immunostainings (Figure S2A). These results indicate that the heating method with its reproducibility and consistency sets a new standard immunostaining protocol for fish.

Antigen retrieval in whole mount preparations has been thought to be difficult without destroying the morphology of the sample. Since the heating method had been successfully applied to whole mount preparations prior to sectioning and antibody staining on sections, we tested the method on whole mount immunostainings. In brief, fixed-embryos after rehydration were heated in 150 mM Tris-HCl at pH 9.0, followed by the standard whole mount fluorescent immunostaining (see details in Materials and Methods). As in Figure 3A, we performed multicolor whole mount fluorescent immunostaining on zebrafish and medaka embryos with the same antibodies under the identical conditions. Strikingly, both GS/Pax6 and PCNA/pH 3 immunostainings clearly showed Mueller glia/amacrine cells and proliferating/dividing mitotic cells, respectively, with a strong enhancement of the signal compared to the standard protocol (Figure 3B (middle and bottom panels) and Figure S2B). Furthermore, the heating method also improved PCNA/pH 3 immunodetection in the entire body, even deep inside tissues, highlighting the other proliferative zones in the hindbrain, optic tectum, telencephalon, and CMZ in both zebrafish and medaka (Figure 3B (top panels) and Figure S2B). Thus, these results demonstrate that our new method has efficiently overcome the problems of heat-mediated antigen retrieval, thereby facilitating efficient and penetrative staining in whole mount preparations fully preserving the overall and tissue morphology.

### Flexible application of the heating method to co-staining with fluorescent proteins (FPs) in transgenic lines and whole-mount *in situ* hybridization

**Figure 4:**
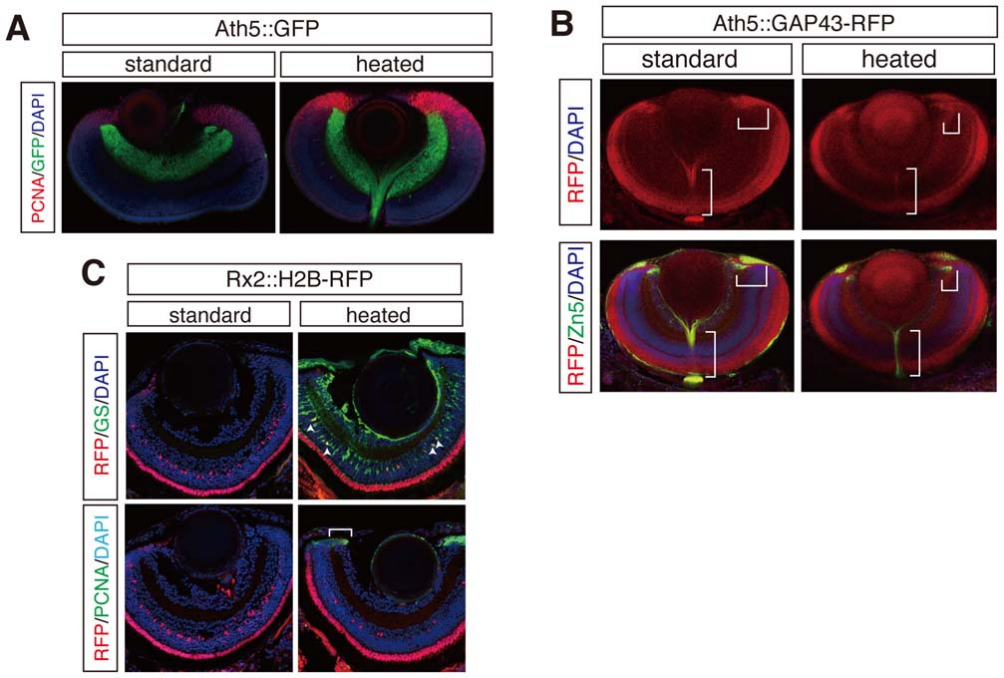
Application of heating method to co-staining with fluorescent proteins (FPs) in transgenic lines. (**A**) PCNA whole-mount immunostaining with Ath5::GFP medaka transgenic line. (**B**) Zn5 whole-mount immunostaining with Ath5::GAP43-RFP zebrafish transgenic line. Note that Zn5 overlaps with RFP-positive optic nerve and retinal progenitor cells (brackets) (**C**) GS and PCNA immunostainings on cryosections of Rx2::H2B-RFP medaka transgenic line. Rx2 dependent H2B-RFP expression clearly co-localizes with GS-positive Mueller glia cells (arrowheads) and PCNA positive retinal progenitor cells in the CMZ (brackets). Nuclei were counterstained with DAPI (blue).

Heating at high temperature can cause denaturation of fluorescent proteins (FPs) with loss of fluorescence and antigenicity [10]. To test whether our heating method is limited by this potential drawback, we performed immunostaining of crysosection and whole mount preparations on transgenic zebrafish and medaka lines. The specific promoters of Ath5 used in the transgenic lines allow to visualize developing retinal ganglion cells (RGCs) with their optic nerve axons by driving the expression of green (GFP) and red fluorescent protein (RFP) respectively (Figure 4A and B, [11]). Applying our new protocol to embryos carrying these transgenes facilitated the detection of GFP and RFP fluorescence at comparable intensity levels as in the untreated control embryos, both in cryosections and in whole mount preparations (Figure 4A and B, and Figure S3A and S3B). The fluorescence of the transgenic GFP itself was sufficient to compare its expression with either PCNA (whole mount) or HuC/D/pH 3 (cryosection) immunostaining in the transgenic line (Figure 4A and Figure S3A). Even the weaker Ath5::GAP43-RFP transgene was efficiently co-detected in immunostanings with the heating method. Using either anti-Zn5 or HuC/D antibody as a marker for RGC, Zn5- and HuC/D-immunostainings on the retinae of transgenic Ath5::GAP43-RFP embryos clearly showed the overlap and co-stain with the RFP-positive RGCs (or their progenitors near the CMZ, see brackets in Figure 4B) in whole mount preparations and cryosections, respectively (Figure 4B and Figure S3B, see brackets and arrowheads). The same results were also obtained in immunostainings of cryosections with the transgenic medaka line Rx2::histone H2B (H2B)-RFP, which labels retinal progenitor and Mueller glia and photoreceptor cells [11]. Immunostainings using either anti-GS, or PCNA antibodies showed co-localization between GS- and PCNA-antibody staining and the H2B-RFP-positive cells in Mueller glia and retinal progenitor cells (Figure 4C, arrowheads and a bracket). Thus, our method is gentle enough to allow the co-detection of genetically encoded fluorescent proteins simultaneous with immunostaining in transgenic embryos, facilitating co-localization studies to rapidly validate the proper localization of fluorescent fusion proteins.

Whole-mount *in situ* hybridization (WISH) has already been combined with immunostaining. To test whether the heat retrieval of antigens could further improve the standard protocols, we performed the fluorescent WISH of medaka using an Rx2 probe, followed by either GS or PCNA immunostaining. While with the standard protocol only rather weak signals for GS and PCNA were detected, heating of the embryos significantly improved the staining resulting in bright and clear signals, showing the overlap with Rx2 mRNA expression in Mueller glia and retinal progenitor cells (Figure S3C, insets). Again, this combination improved the standard protocol for comparison of expression patterns of mRNA and proteins within the same sample.

### Conclusions

Here we have established a novel heating method for fluorescent immunostaining that is simple and efficient and provides universal conditions for the vertebrate model organisms medaka and zebrafish. Several methods to retrieve antigen such as enzymatic and heating approaches depending on different buffers and instruments (such as micro-wave or steaming autoclave) have been previously described [12], [13]. However, those methods often led to the loss or even destruction of the samples [12], [13]. Even though the heating step we introduced sounds drastic, it was rather gentle and preserved organ and tissue morphology both at embryonic and juvenile stages. For application at blasutla and gastrula stages a slight modification of the heat treatment (shorter, lower temperature) is suggested. While the method drastically improves the retrieval of many antigens, some temperature-sensitive epitopes may not be detectable possibly due the masking of epitopes during the heating (e. g. Zn5 and Zpr1; see Figure 2).

Our method neither requires special instruments nor specifically adapted buffers for each antibody and (with the exception mentioned above) dramatically exceeds the results of the standard protocol using one universal set of conditions for all antibodies tested. Furthermore, the combination of single or multi-color immunostaing with a wide range of other applications (such as co-stainings with FPs, and also WISH) is easily possible. Practical and efficient at high dilutions of antibody, our method is enabling to cut costs and save precious material. Finally, the method efficiently addresses the long-standing problem on optimization of immunostaining in fish community as well as general pathological analyses, and is likely applicable in other species.

## Materials and Methods

### Ethics statement

All fish are housed in the fish facility of our laboratory, which was built according to the local animal welfare standards (Tierschutzgesetz §11, Abs. 1, Nr. 1) and in accordance with European Union animal welfare guidelines. The facility is under the supervision of the local representative of the animal welfare agency. No animal experiments were performed. Embryos of medaka (*Oryzias latipes*) and zebrafish (*Danio rerio*) were used exclusively at stages prior to hatching (not considered as animals according to German law and European union regulations). Zebrafish and medaka were raised and maintained as described previously [11], [14]. The following strains were used for zebrafish embryos: wild type WIK/AB and the transgenic line Ath5::GAP43-RFP [14], [15]. The following strains were used for medaka embryos: The Cab wild type strain, the Heino albino strain, and the transgenic lines Ath5::GFP and Rx2::H2B-RFP (The same construct as in [11] just replaced by H2B-RFP, L. Centanin, the line unpublished) generated in our lab [11], [16], [17].

### Treatment and preparation of fish embryos

Fertilized eggs of medaka and zebrafish were routinely collected as described previously [11], [14], [17]. The embryos were incubated at 28°C until 6 days (zebrafish) or 7 days (for medaka) post fertilization. Except for the Heino strain, all the strains and transgenic lines of both medaka and zebrafish embryos were incubated with 5×PTU to prevent pigmentation. The embryos were fixed with 4%PFA at 4°C overnight. For fluorescent immunostaining of either cryosecctions or whole mount embryos, fixed embryos were dechorionated (for medaka) and equilibrated in 1×PTw (1×PBS at pH 7.3, 0.1% Tween), followed by appropriate steps including the heating method for either cyrosection or whole mount immunostaining (see below for details). Prior to perform combined whole mount *in situ* hybridization and immunostaining, dechorionated medaka embryos were washed with 1×PTw for 5 min several times, and then stored in 100% MeOH at −20°C at least two days. Those embryos were subjected to fluorescent whole mount *in situ* hybridization including the heating method and immunostaining (see below for details).

### The heating method prior to cryosection and whole mount immunostaining

Fixed embryos were rehydrated with 1×PTw for 10 min three times, and then cryoprotected in 30% sucrose at 4°C at least overnight. For the heating procedure, the cryoprotected embryos were incubated in 150 mM Tris-HCl at pH 9.0 for 5 min, followed by heating at 70°C for 15 min. After heating, the embryos were re-cryoprotected in 30% sucrose at 4°C overnight. Those cyroprotected embryos were embedded in the mold with the freezing medium (Jung, Leica microsystems), sectioned (18 µm thickness) by the cryotome (Leica CM3050S). Cryosections were mounted on the slide (Superfrost plus, Thermo scientific) and dried for at least 3 h and subjected to immunostaining. For whole mount immunostaining, either fixed or cryoprotected embryos were equilibrated in 1×PTw for 10 min three times, and then subjected to the heating procedure. Since re-cryoprotection was not required for whole mount immunostaining, embryos were directly washed after the heating treatment in 1×PTw for 2×10 min and then washed in dH_2_O for 2×5 min. Subsequently, to enhance tissue permeabilization, for whole mount immunostaining all embryos were treated with acetone for 20 min at −20°C After six sequential washes in 1×PTw (5 min each), the embryos were subjected to whole mount immunostaining (see below).

### Fluorescent immunostaining with either cryosection or whole mount embryos

For immunostaining on cryosections, the slides were equilibrated in 1×PTw for 3×5 min, without shaking, and then blocked with 10% sheep serum (SIGMA, S-2263) in 1×PTw for 1 h at room temperature. After blocking, the slides were incubated with the respective primary antibodies at 4°C overnight (henceforth, unless otherwise stated, all primary and secondary antibodies' dilution rate was fixed as the universal condition: 1∶500) and then washed in 1×PTw for 3×10 min with minimal shaking (≦100 rpm). For secondary antibody incubation, the slides were incubated with the respective antibodies at 37°C for 1.5 h, and then washed in 1×PTw for 2×10 min. After the washes, DAPI (SIGMA, D9564) staining was performed for 5 min at room temperature (final concentration; 100 µg/ml, 1∶1000 dilution), followed by several washes in 1×PTw. For preservation the slides were overlaid with 100 µl of 60% glycerol covered with a coverslip, sealed with the nail polish and stored at −20°C until use.

For immunostaining of whole mount embryos after the acetone treatment, the embryos were sequentially washed in dH_2_O for 2×5 min and 1×PTw for 2×5 min, and then incubated in the blocking buffer (B-buffer) (10% sheep serum, 0.8% Triton X-100, 1% BSA in 1×PTw) for 3 h at 4°C. After blocking, the embryos were incubated with the respective primary antibodies in the incubation buffer (I-buffer) (1% sheep serum, 0.8% Triton X-100, 1% BSA in 1×PTw ) at 4°C for three days gently agitated on a turning wheel. To remove residual primary antibody, embryos were sequentially washed with PBS-TS (10% sheep serum, 1% Triton X-100 in 1×PBS) for 3×1 h, with PBS-T (1% Triton X-100 in 1×PBS) for 2×10 min, and again with PBS-TS for 2×1 h. The embryos were incubated with secondary antibodies and DAPI (1∶500 dilution) in the dark for two and half days, and then washed with PBS-TS for 3×1 h and with 1×PTw for 2×1 h. To take pictures, embryos were embedded in 1% low-melting agarose on a glass bottom culture dish (MatTek corporation). Further information on the antibodies used is given in Figure 2. Secondary antibodies were Alexa Fluor 488 goat anti-rabbit and anti-mouse IgG (Invitrogen), Alexa Fluor 546 goat anti-mouse IgG (Invitrogen), Dylight 549 goat anti-rabbit IgG (Jackson immuno research), and Cy5 goat anti-rabbit IgG (Millipore). All images were acquired by confocal microscopy (Leica TCS SPE), analyzed and adjusted with the software Fiji, transformed into TIFF image by Photoshop (Adobe systems).

### Combined fluorescent whole mount *in situ* hybridization and immunostaining

Fluorescent whole mount *in situ* hybridization was essentially carried out as described previously [11]. Briefly, after rehydration by sequential washing with gradient dilution of MeOH, the embryos were heated with 150 mM Tris-HCl at pH 9.0 as in the immunostaining. The heated embryos were treated with Proteinase K (10 µg/ml in PTw) for 30 min (for ≧St.37 of medaka), and then washed with 1×PTw several times, followed by prehybridization with hybridization-mix at 65°C for 2 h. For *in situ* hybridization, the embryos were incubated in 180 µl hybridization-mix containing 20 µl of DIG-labeled anti-sense Rx2 RNA probe at 65°C overnight. For primary antibody incubation, the embryos were blocked with TNB (100 mM Tris-HCl at pH 7.5, 150 mM NaCl, 0.1% Tween, 2% blocking reagent (Roche)) for 2 h at room temperature, and then incubated with I-buffer containing anti-DIG-POD antibody (Roche) in combination with either anti-GS antibody (Chemicon) or anti-PCNA antibody (Santa Cruz) for two days at 4°C TSA-Plus Cyanine 3 system (Perkin Elmer) was used to detect Rx2 mRNA expression according to the manufacture's instruction. The embryos were then incubated with I-buffer containing anti-mouse Alexa Fluor 488 antibody and DAPI (1∶500) for two days at 4°C. Washing was performed with TBS-T (100 mM Tris-HCl at pH 7.5, 150 mM NaCl, 0.1% Tween) for 10 min at least 5 times after primary and secondary antibody incubation as well as TSA reaction. For taking pictures, embryos were mounted as described above for immunostaining.

## Supporting Information

Figure S1 **Comparable and improved fluorescent immunostainings of cryosections by the heating method.** (**A**) Comparable fluorescent immunostainings of cryosections with or without the heating method in zebrafish and medaka. Note that the heating method as well as standard protocol fully preserved the retinal morphology. (**B**) Immunostainings improved by the heating method. HuC/D (bracket), phospho-histone H3 (pH 3) (arrowheads), and GFAP (brackets) immunostainings were strongly improved as compared to standard protocol in both zebrafish and medaka. (**A**) and (**B**) Rhodopsin (brackets) and Recoverin; photoreceptor cell layer. Pax6; amacrine cells. Islet1; retinal ganglion and neuronal cells in the inner nuclear layer. HuC/D; retinal ganglion and amacrine cell layers. pH 3; mitotic retinal progenitor cells. GFAP; Mueller glia cells. Nuclei were counterstained with DAPI (blue).
(TIF)

Figure S2 **Multiple fluorescent immunostaining of cryosections and whole mount embryos by the heating method in medaka.** (**A**) and (**B**) PCNA/pH 3 and GS/Pax6 fluorescent immunostainings of medaka cryosections (in **A**) and whole mount embryos (in **B**) with or without the heating method. (**B**) Top panels; Z-stack image of dorsal view of whole mount medaka. Brackets indicate proliferative zones of optic tectum, telencephalon, and CMZ. Asterirsks denote PCNA-positive cells in the tectum neuropil. Bottom panels; coronal optic sections of medaka retina. L; lens. Nuclei were counterstained with DAPI (blue).
(TIF)

Figure S3 **Application of heating method to co-staining with fluorescent proteins (FPs) in transgenic lines and whole-mount** ***in situ*** **hybridization.** (**A**) HuC/D and pH 3 immunostainings of cryosections with Ath5::GFP medaka transgenic line. The heating method sufficiently retained GFP fluorescence after heating. GFP-positive retinal ganglion cells co-stained with HuC/D-positive retinal ganglion cells (a bracket) and pH 3-positive mitotic dividing cells (arrowheads). (**B**) HuC/D immunostaining of cryosections with Ath5::GAP43-RFP (RFP) zebrafish transgenic line. Note that fluorescent signal was still strong enough to detect after heating. (**C**) Whole mount *in situ* hybridization of medaka by uing antisense Rx2 probe in combination with either GS or PCNA immunostaining. Note that Rx2 co-stained with GS-positive Mueller glia cells (arrowheads) and PCNA-positive retinal progenitor cells at CMZ (brackets). Insets denote higher magnification of the overlap regions. Nuclei were counterstained with DAPI (blue).
(TIF)
