# Supplementary figures and images for: One for All—A Highly Efficient and Versatile Method for Fluorescent Immunostaining in Fish Embryos
Source: PLoS One. 2011 May 13;6(5):e19713. doi: 10.1371/journal.pone.0019713 (PMC3094454; doi:10.1371/journal.pone.0019713)

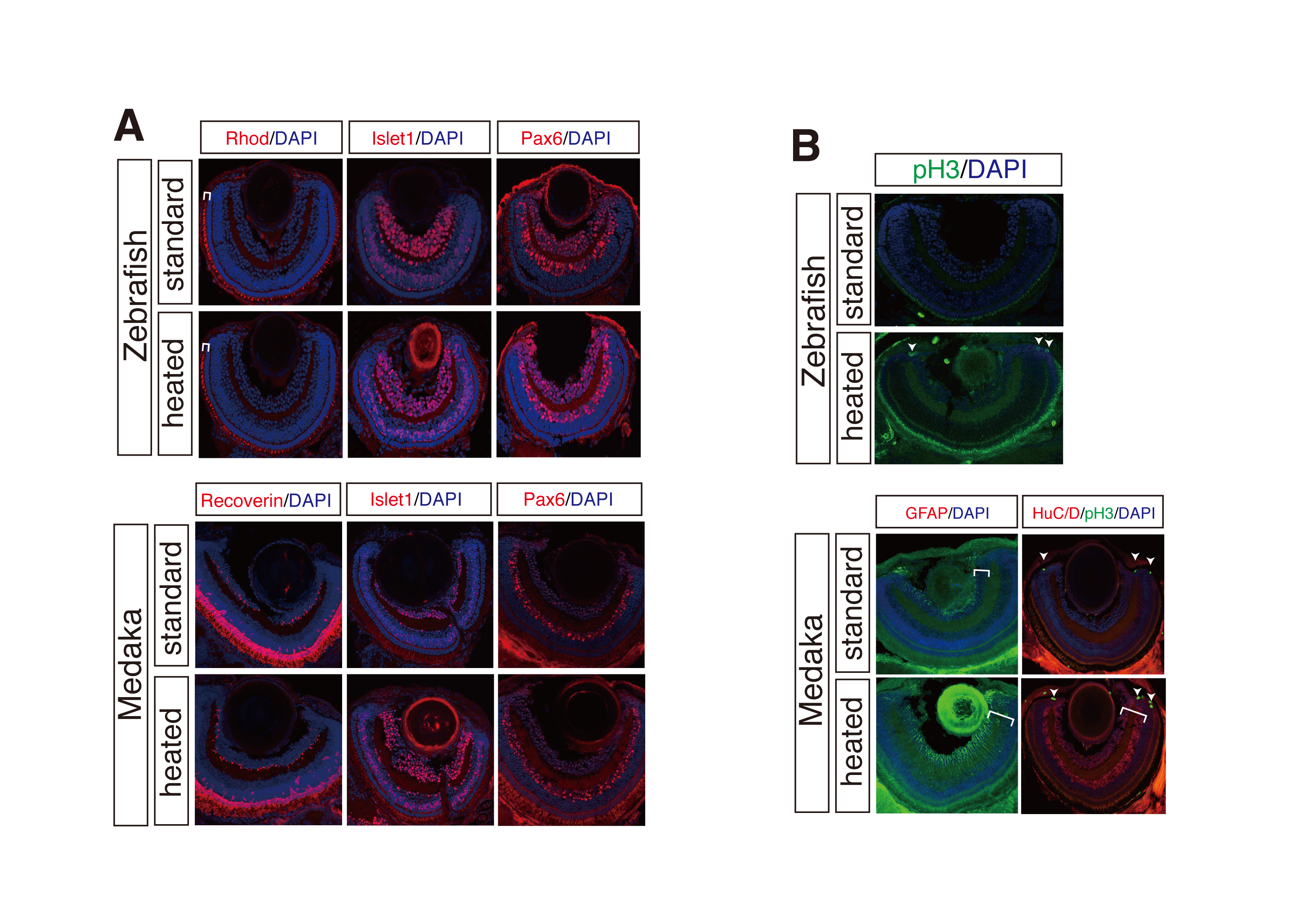

Supplement: Figure S1 — Comparable and improved fluorescent immunostainings of cryosections by the heating method. (A) Comparable fluorescent immunostainings of cryosections with or without the heating method in zebrafish and medaka. Note that the heating method as well as standard protocol fully preserved the retinal morphology. (B) Immunostainings improved by the heating method. HuC/D (bracket), phospho-histone H3 (pH 3) (arrowheads), and GFAP (brackets) immunostainings were strongly improved as compared to standard protocol in both zebrafish and medaka. (A) and (B) Rhodopsin (brackets) and Recoverin; photoreceptor cell layer. Pax6; amacrine cells. Islet1; retinal ganglion and neuronal cells in the inner nuclear layer. HuC/D; retinal ganglion and amacrine cell layers. pH 3; mitotic retinal progenitor cells. GFAP; Mueller glia cells. Nuclei were counterstained with DAPI (blue). (TIF) [file pone.0019713.s001.tif]

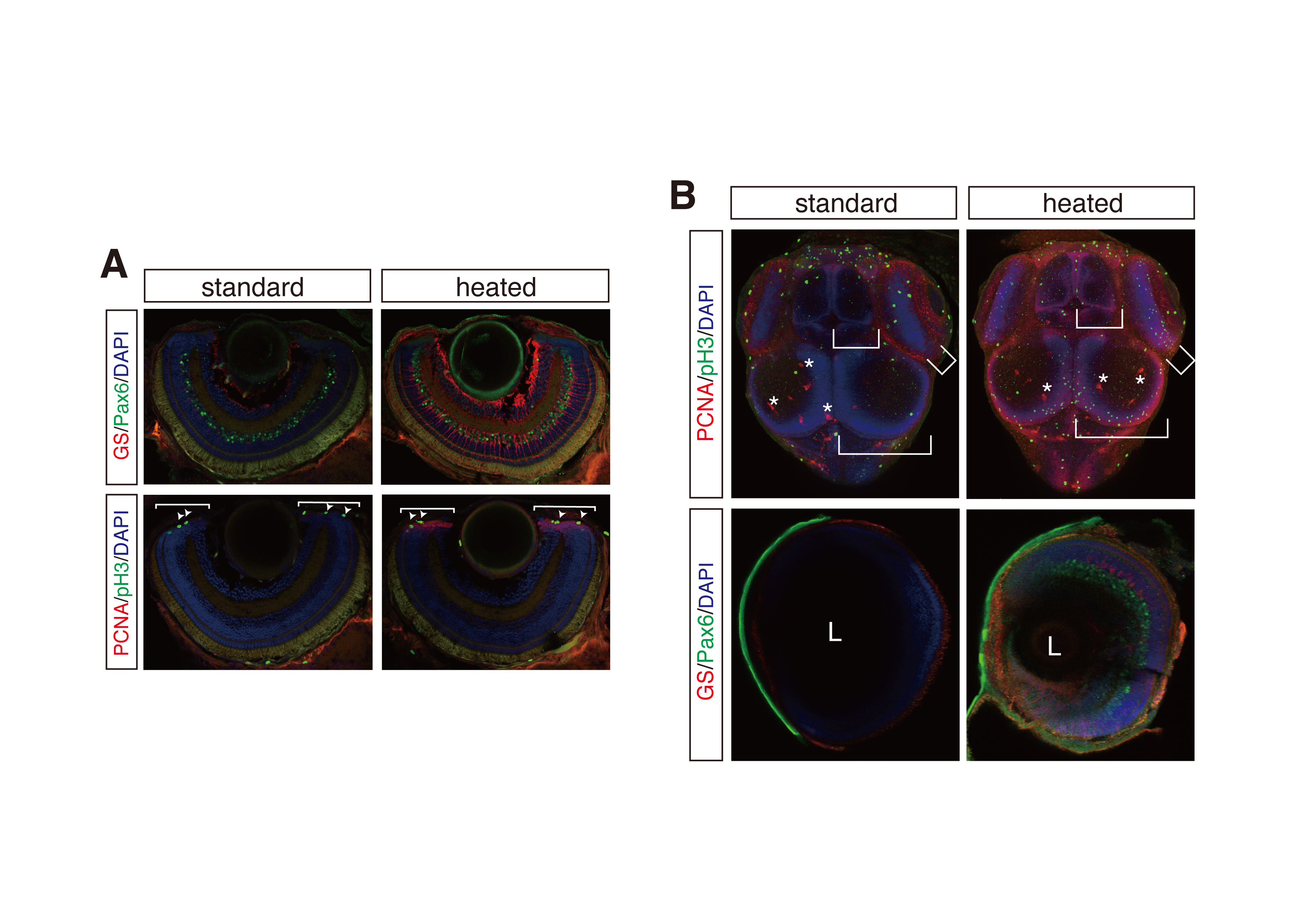

Supplement: Figure S2 — Multiple fluorescent immunostaining of cryosections and whole mount embryos by the heating method in medaka. (A) and (B) PCNA/pH 3 and GS/Pax6 fluorescent immunostainings of medaka cryosections (in A) and whole mount embryos (in B) with or without the heating method. (B) Top panels; Z-stack image of dorsal view of whole mount medaka. Brackets indicate proliferative zones of optic tectum, telencephalon, and CMZ. Asterirsks denote PCNA-positive cells in the tectum neuropil. Bottom panels; coronal optic sections of medaka retina. L; lens. Nuclei were counterstained with DAPI (blue). (TIF) [file pone.0019713.s002.tif]

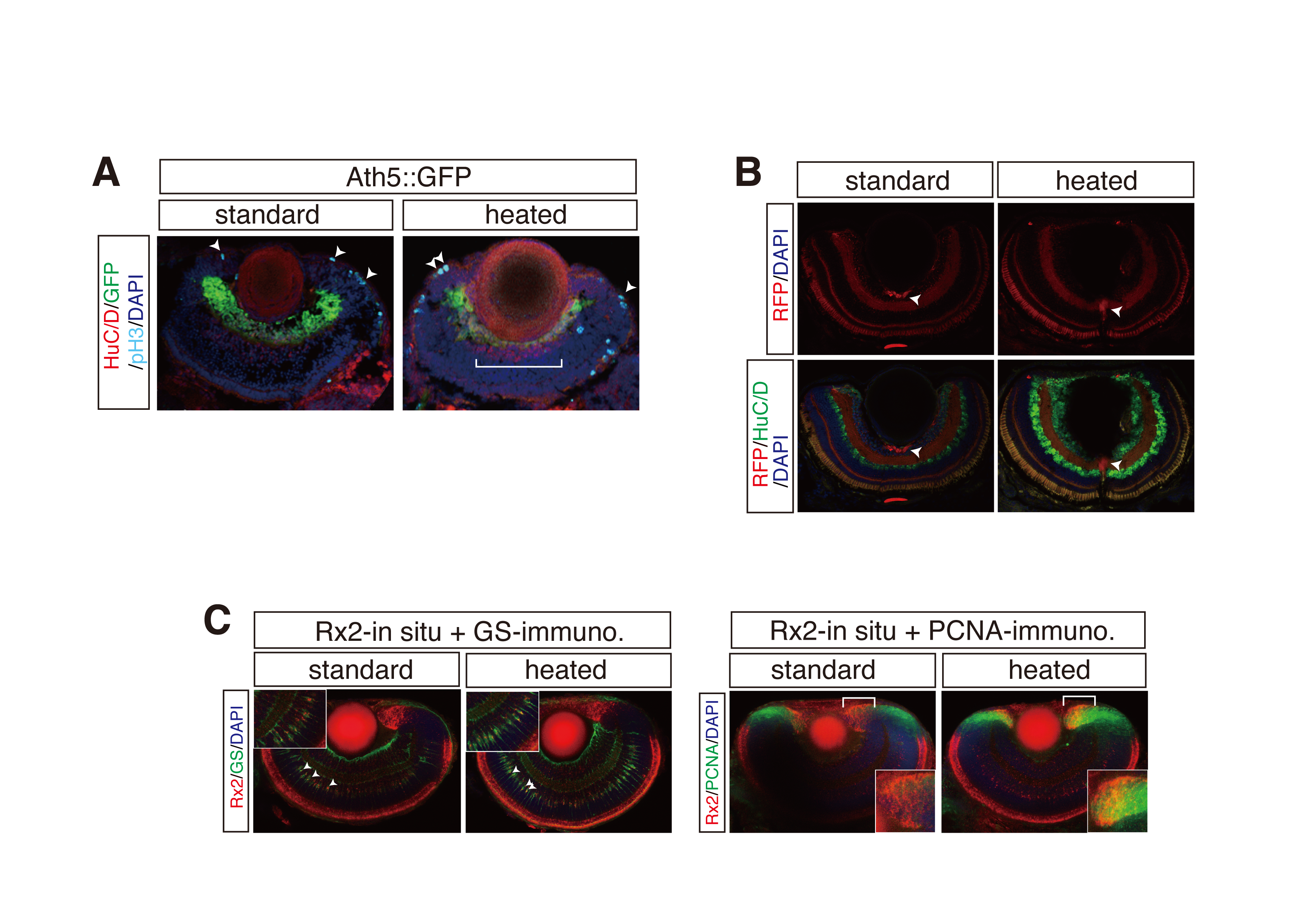

Supplement: Figure S3 — Application of heating method to co-staining with fluorescent proteins (FPs) in transgenic lines and whole-mount in situ hybridization. (A) HuC/D and pH 3 immunostainings of cryosections with Ath5::GFP medaka transgenic line. The heating method sufficiently retained GFP fluorescence after heating. GFP-positive retinal ganglion cells co-stained with HuC/D-positive retinal ganglion cells (a bracket) and pH 3-positive mitotic dividing cells (arrowheads). (B) HuC/D immunostaining of cryosections with Ath5::GAP43-RFP (RFP) zebrafish transgenic line. Note that fluorescent signal was still strong enough to detect after heating. (C) Whole mount in situ hybridization of medaka by uing antisense Rx2 probe in combination with either GS or PCNA immunostaining. Note that Rx2 co-stained with GS-positive Mueller glia cells (arrowheads) and PCNA-positive retinal progenitor cells at CMZ (brackets). Insets denote higher magnification of the overlap regions. Nuclei were counterstained with DAPI (blue). (TIF) [file pone.0019713.s003.tif]
